# Comparison of renin–angiotensin–aldosterone system inhibitors with other antihypertensives in association with coronavirus disease-19 clinical outcomes

**DOI:** 10.1186/s12879-021-06088-6

**Published:** 2021-06-05

**Authors:** Yihienew M. Bezabih, Alemayehu Bezabih, Endalkachew Alamneh, Gregory M. Peterson, Woldesellassie Bezabhe

**Affiliations:** 1 Arsi University College of Health Sciences, Arsi University, P. O. Box, 04, Assela, Ethiopia; 2 École Nationale Vétérinaire, Agroalimentaire et de L’alimentation, Nantes-Atlantique, BIOEPAR (UMR1300 INRA/ONIRIS), Atlanpole-Chantrerie CS-40706 44307, Nantes Cedex 3, France; 3 School of Pharmacy and Pharmacology, University of Tasmania, Private Bag 26, Hobart, TAS 7001 Australia

**Keywords:** RAAS inhibitors, COVID-19, Coronavirus, Angiotensin, Clinical outcome, ACE2

## Abstract

**Background:**

Reports on the effects of renin–angiotensin–aldosterone system (RAAS) inhibitors on the clinical outcomes of coronavirus disease-19 (COVID-19) have been conflicting. We performed this meta-analysis to find conclusive evidence.

**Methods:**

We searched published articles through PubMed, EMBASE and medRxiv from 5 January 2020 to 3 August 2020. Studies that reported clinical outcomes of patients with COVID-19, stratified by the class of antihypertensives, were included. Random and fixed-effects models were used to estimate pooled odds ratio (OR).

**Results:**

A total 36 studies involving 30,795 patients with COVID-19 were included. The overall risk of poor patient outcomes (severe COVID-19 or death) was lower in patients taking RAAS inhibitors (OR = 0.79, 95% CI: [0.67, 0.95]) compared with those receiving non-RAAS inhibitor antihypertensives. However, further sub-meta-analysis showed that specific RAAS inhibitors did not show a reduction of poor COVID-19 outcomes when compared with any class of antihypertensive except beta-blockers (BBs). For example, compared to calcium channel blockers (CCBs), neither angiotensin-I-converting enzyme inhibitors (ACEIs) (OR = 0.91, 95% CI: [0.67, 1.23]) nor angiotensin-II receptor blockers (ARBs) (OR = 0.90, 95% CI: [0.62, 1.33]) showed a reduction of poor COVID-19 outcomes. When compared with BBs, however, both ACEIs (OR = 0.85, 95% CI: [0.73, 0.99) and ARBs (OR = 0.72, 95% CI: [0.55, 0.94]) showed an apparent decrease in poor COVID-19 outcomes.

**Conclusions:**

RAAS inhibitors did not increase the risk of mortality or severity of COVID-19. Differences in COVID-19 clinical outcomes between different class of antihypertensive drugs were likely due to the underlying comorbidities for which the antihypertensive drugs were prescribed, although adverse effects of drugs such as BBs could not be excluded.

**Supplementary Information:**

The online version contains supplementary material available at 10.1186/s12879-021-06088-6.

## Background

The effect of renin–angiotensin–aldosterone system (RAAS) inhibitors on the clinical outcomes of coronavirus disease-19 (COVID-19) is of great interest [1]. This is because RAAS blockers, one of the most commonly prescribed antihypertensive drug groups, were previously reported to have some interactions with the pathophysiology of severe acute respiratory syndrome coronavirus 2 (SARS-CoV-2) [1, 2].

Experimental studies have shown that blockage of RAAS by either angiotensin-I-converting enzyme inhibitors (ACEIs) or angiotensin-II receptor blockers (ARBs) substantially upregulates the expression of host angiotensin-converting enzyme 2 (ACE_2_) [3], a transmembrane enzyme used by SARS-CoV-2 as a receptor to enter and infect cells [4]. On the other hand, ACE_2_ catalyzes the degradation of potentially harmful angiotensin-II to a vasodilator angiotensin (1–7), which has antiarrhythmic and cardioprotective effects [2, 3]. In addition, RAAS inhibitors may also prevent some complications of COVID-19, such as hypokalaemia. Hence, despite concerns that overexpression of ACE_2_ with RAAS inhibitors could facilitate infection of tissues by SARS-CoV-2, these drugs could also have a therapeutic role.

Recent studies on the effects of RAAS inhibitors (ACEIs and ARBs) on the clinical outcomes of patients with COVID-19 have reported conflicting results, ranging from a decrease in mortality [5, 6], no effect [7–10] or even an increase in mortality [11]. Even previous meta-analysis studies had conflicting findings that reported either a decrease [12–14] or an increase [15] in mortality with RAAS inhibitors. These varying effects on mortality may not be caused by the drugs themselves and could be related to the underlying comorbidities that guided the antihypertensive drug selection (e.g. beta-blockers (BBs) for a hypertensive patient with angina). This bias could partially be avoided by performing multiple sub-meta-analysis comparing one specific class of antihypertensive to another antihypertensive class. This permits a fair comparison of antihypertensive drugs with similar indication and helps us to keep compelling comorbidities in mind when comparing class of drugs with totally different indications (e.g. BBs for heart failure with systolic dysfunction versus thiazides for hypertension without this comorbidity [16]). As no prior meta-analysis made such analysis, we compared the of risk developing poor COVID-19 clinical outcomes among the five specific classes of antihypertensives: (ACEIs, ARBs, BBs, calcium channel blockers (CCBs), and thiazides). In addition, this updated systematic review and meta-analysis included the most recent studies to estimate the overall risk of poor COVID-19 outcomes in patients receiving RAAS inhibitors compared to those receiving non-RAAS inhibitor antihypertensive agents.

## Methods

This study was conducted following the Preferred Reporting Items for Systematic Reviews and Meta-Analyses (PRISMA) 2009 checklist [17] (Supplementary Table 1 (Table S1)).

### Data sources and search terms

**Fig. 1:**
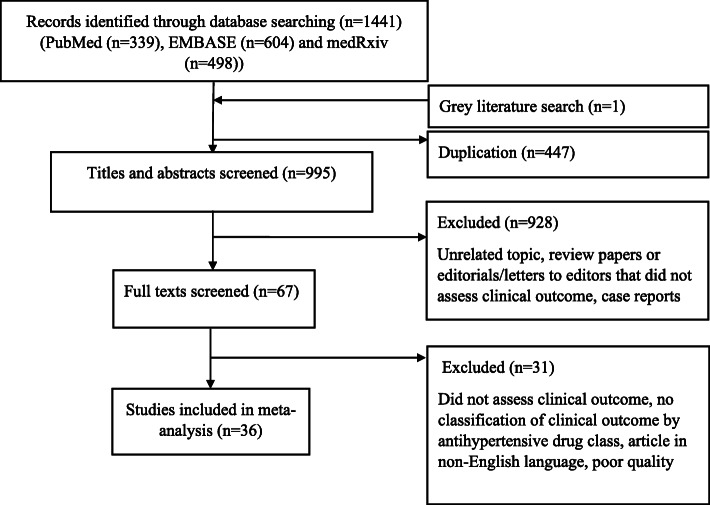
Flow chart showing the selection of articles for the meta-analysis

We searched PubMed, EMBASE and medRxiv preprint server to identify potentially relevant articles published between 5 January 2020 to 3 August 2020. A Google grey literature search was also performed to find additional articles that may have not been indexed. We used three main search keywords: (1) clinical outcome OR death OR mortality, (2) angiotensin and (3) COVID. These key words were combined with Boolean operators to make the following search term: (((((clinical outcome) OR death) OR mortality)) AND angiotensin) AND COVID. We found 339 and 604 articles indexed in PubMed and EMBASE, respectively (Fig. 1). We also found 498 articles from medRxiv preprint server and one article from manual search (Fig. 1). Two authors (Y. B., W. B.) selected studies by screening titles and abstracts. A third author (E. A) served as a mediator to reach a consensus for discrepancies.

### Study definitions

RAAS inhibitors in this study refer to only ACEIs and ARBs whereas non-RAAS inhibitors include CCBs, BBs and thiazide diuretics. Severe COVID-19 refers to the presence of any of the following: respiratory rate ≥ 30 breaths/minute, oxygen saturation at rest ≤93%, oxygenation index [partial pressure of arterial oxygen (PaO2)/ percentage of inspired oxygen (FiO2)] ≤300 mmHg, respiratory or other organ failure, mechanical ventilation, shock, or intensive care unit treatment [18]. We used the term ‘poor clinical outcome’ to indicate the presence of either severe COVID-19 or death. Main meta-analysis refers to the overall comparison of RAAS inhibitors to non-RAAS inhibitor drugs whereas sub-meta-analyses were comparison between specific class of drugs within the above two major groups of antihypertensives (e.g. ACEIs to CCBs).

### Outcome of interest

The main outcome of interest was the overall risk of having poor clinical outcomes in patients infected with COVID-19 while receiving RAAS inhibitors, compared with those taking other antihypertensive agents. The secondary outcome was the risk of severe COVID-19 or death in patients receiving a specific RAAS inhibitor (e.g. ACEIs) compared with those receiving other classes of antihypertensives.

### Study selection: inclusion and exclusion criteria

Studies that reported the clinical outcomes of COVID-19 patients stratified by class of antihypertensive drug therapy (treated group on RAAS inhibitors and control group on non-RAAS inhibitors) were included. Cohort (prospective or retrospective) studies, clinical trials, case series studies and editorials/letters that assessed COVID-19 clinical outcomes for patients taking RAAS inhibitors versus non-RAAS inhibitors were included. The included papers were either published (including preprint servers) or accepted original articles written in English. We excluded review papers and case reports. In addition, studies that compared COVID-19 clinical outcomes in two groups where the treated group were taking RAAS inhibitors whereas the control group were not taking any form of antihypertensive (e.g. hypertension requiring only dietary management) were ineligible. This was to have comparable groups in terms of the severity level of the comorbidity.

### Data extraction and quality control

In each study, the total number of patients taking RAAS inhibitors or other class (es) of antihypertensives was recorded. Then, for each antihypertensive class exposure, the total number of patients with a poor clinical outcome (severe COVID-19 or death) versus those with a good outcome (non-severe COVID-19 and survival) were recorded. In addition, year, design of study and nature of comorbidities were also documented (Table 1).

**Table 1:** General characteristics of enrolled patients

**Study**	**Study design**	**Comorbidity**	**Drug class**	**Survivors**	**Non-survivors**	**Total (survivors + non-survivors)**	**% poor outcome per drug class**
Zhang et al., 2020 [6]	Retrospective cohort	HTN	ACEI/ARBs	181	7	188	3.7%
			Non-ACEI/ARBs	848	92	940	9.8%
IP et al., 2020 [19]	Retrospective cohort	HTN	ACEI/ARBs	323	137	460	29.8%
			Non-ACEI/ARBs	407	262	669	39.2%
Khera et al., 2020 [20]	Retrospective cohort	HTN	ACEIs	2,042	319	2,361	13.5%
			ARBs	1,881	345	2,226	15.5%
			Non-ACEI/ARBs	2,880	466	3,346	13.9%
Richardson et al., 2020 [21]	Case series	HTN	ACEIs	113	55	168	32.7%
			ARBs	170	75	245	30.6%
Tan et al., 2020 [22]	Retrospective cohort	HTN	ACEI/ARBs	29	0	29	0.0%
			Non-ACEI/ARBs	46	11	57	19.3%
Andrea et al., 2020 [23]	Retrospective cohort	HTN, HF, CAD, DM, CKD	ACEIs	21	14	35	40.0%
			ARBs	26	7	33	21.2%
			BBs	29	21	50	42.0%
			CCBs	16	9	25	36.0%
			Thiazides	12	4	16	25.0%
Xian Zhou et al., 2020 [24]	Retrospective cohort	HTN, HF, CAD, DM, CKD	ACEIARB	13	2	15	13.3%
			Non-ACE/ARB	16	5	21	23.8%
Feng Zhou et al., 2020 [5]	Retrospective cohort	HTN, CAD	ACEI/ARB	836	70	906	7.7%
			Non-ACEI/ARB	1,540	272	1,812	15.0%
Pan et al., 2020 [25]	Retrospective cohort	HTN	ACEI/ARB	37	4	41	9.8%
			Non-ACE/ARB	178	63	241	26.1%
Cannata et al., 2020 [26]	Prospective cohort	Not mentioned	ACEI/ARB	49	7	56	12.5%
			Non-ACE/ARB	185	39	224	17.4%
Lam et al., 2020 [27]	Prospective cohort	HTN, CAD, DM, CKD	ACEI/ARB	277	58	335	17.3%
			Non-ACEI/ARB	217	62	279	22.2%
Selcuk et al., 2020 [28]	Retrospective cohort	HTN, HF, CAD, DM, CKD	ACEI/ARB	43	31	74	41.9%
			Non-ACE/ARB	35	4	39	10.3%
Amat-Santos et al., 2020 [29]	Randomized clinical trial	HTN	ACEI/ARB	3	2	5	40.0%
			Non-ACE/ARB	4	2	6	33.3%
Felice et al., 2020 [30]	Prospective cohort	HTN	ACEIs	32	8	40	20.0%
			ARBs	35	7	42	16.7%
**Study**	**Study design**	**Comorbidity**	**Drug class**	**Non severe COVID-19**	**Severe COVID-19**	**Total (severe and non-severe COVID-19)**	**% severe COVID-19 per drug class**
Reynolds et al., 2020 [7]	Retrospective cohort	HTN	ACEIs	445	139	584	23.8%
			ARBs	468	161	629	25.6%
			BBs	582	210	792	26.5%
			CCBs	697	253	950	26.6%
			Thiazides	399	116	515	22.5%
Li et al., 2020 [8]	Retrospective cohort	HTN	ACEIs	9	3	12	25.0%
			ARBs	13	11	24	45.8%
			BBs	6	8	14	57.1%
			CCBs	89	79	168	47.0%
Feng et al., 2020 [31]	Prospective cohort	HTN	ACEIs	7	1	8	12.5%
			ARBs	23	4	27	14.8%
			Non-ACEI/ARBs	35	27	62	43.6%
Yang et al., 2020 [32]	Retrospective cohort	HTN	ACEI/ARBs	28	15	43	34.9%
			Non-ACEI/ARBs	48	35	83	42.2%
Meng et al., 2020 [9]	Retrospective cohort	HTN	ACEI/ARBs	13	4	17	23.5%
			Non-ACEI/ARBs	13	12	25	48.0%
Gao et al., 2020 [33]	Retrospective cohort	HTN	ACEI/ARBs	109	74	183	40.4%
			Non-ACEI/ARBs	348	179	527	34.0%
Hu et al., 2020 [34]	Retrospective cohort	HTN	ACEI/ARBs	37	28	65	43.1%
			Non-ACEI/ARBs	51	33	84	39.3%
Liu et al., 2020 [35]	Retrospective cohort	HTN	ACEIs	1	1	2	50.0%
			ARBs	7	3	10	30.0%
			BBs	4	3	7	42.9%
			CCBs	8	18	26	69.2%
			Thiazides	3	0	3	0.0%
Zeng et al., 2020 [36]	Retrospective cohort	HTN	ACEI/ARBs	13	15	28	53.6%
			Non-ACEI/ARBs	32	15	47	31.9%
Bravi et al., 2020 [37]	Retrospective cohort	HTN	ACEIs	107	144	251	57.4%
			ARBs	86	142	228	62.3%
Dauchet et al., 2020 [38]	Retrospective cohort	CVD	ACEIs	14	13	27	48.2%
			ARBs	8	21	29	72.4%
Feng Zhichao et al., 2020 [39]	Retrospective cohort	HTN	ACEI/ARBs	15	1	16	6.3%
			Non-ACEI/ARBs	33	16	49	32.7%
Mancia et al., 2020 [40]	Case control study	CVD	ACEIs	1,305	197	1,502	13.1%
			ARBs	1,227	167	1,394	12.0%
			BBs	1,556	270	1,826	14.8%
			CCBs	1,230	216	1,446	14.9%
			Thiazides	991	113	1,104	10.2%
Yan et al., 2020 [41]	Case control study	CVD	ACEIs	4	14	18	77.8%
			ARBs	58	93	151	61.6%
			BBs	9	47	56	83.9%
			CCBs	230	158	388	40.7%
			Thiazides	14	21	35	60.0%
Senkal et al., 2020 [42]	Retrospective cohort	HTN, HF, CAD, DM, CKD	ACEIs	41	11	52	21.2%
			ARBs	36	16	52	30.8%
			Non-ACEI/ARBs	30	22	52	42.3%
Liabeuf et al., 2020 [43]	Retrospective cohort	HTN, HF, CAD, DM, CKD	ACEI/ARBs	44	52	96	54.2%
			BBs	36	37	73	50.7%
			CCBs	30	27	57	47.4%
			Thiazides	28	30	58	51.7%
Sardu et al., 2020 [44]	Prospective cohort	HTN	ACEIs	14	10	24	41.7%
			ARBs	12	9	21	42.9%
			CCBs	10	7	17	41.2%
Xiulan Liu et al., 2020 [45]	Retrospective cohort	HTN	ACEI/ARBs	20	18	38	47.4%
			CCBs	22	16	38	42.1%
Lopez-Otero et al., 2020 [46]	Retrospective cohort	HTN, CAD, DM	ACEIs	23	6	29	20.7%
			ARBs	43	7	50	14.0%
Golpe et al., 2020 [47]	Retrospective cohort	HTN, HF, CAD, DM, CKD	ACEIs	20	12	32	37.5%
			ARBs	53	36	89	40.5%
			BBs	24	23	47	48.9%
			CCBs	21	23	44	52.3%
			Thiazides	36	30	66	45.5%
Xu et al., 2020 [48]	Retrospective cohort	HTN, HF, CAD, DM, CKD	ACEI/ARBs	29	11	40	27.5%
			Non-ACEI/ARBs	45	16	61	26.2%
Choi et al., 2020 [49]	Case control study	HTN	ACEI/ARBs	859	33	892	3.7%
			Non-ACEI/ARBs	384	44	428	10.3%
Total				24,759	6,036	30,795	19.6%

*Abbreviations*: *ACEI* angiotensin-I-converting enzyme inhibitors, *ARBs* angiotensin-II receptor blockers, *BBs* beta-blockers, *CAD* coronary artery disease, *CCBs* calcium channel blockers, *CKD* chronic kidney disease, *CVD* cardiovascular diseases, *DM* diabetes, *HF* heart failure, *HTN* hypertension

The Newcastle-Ottawa quality assessment scale (NOS) [50] was used for quality assessment of the included studies (Table S2). Two reviewers (W.B. and E.A.) independently performed the quality assessment and another author (Y.B.) brought consensus during discrepancies. Articles which got a score of less than 7 stars in the NOS were considered poor quality and excluded (Table S2).

### Data analysis

A random-effects meta-analysis using the DerSimonian and Laird method [51] was used to estimate pooled odds ratio (OR) whenever the heterogeneity (I^2^) was above 25% and the fixed effects model (Mantel-Haenszel) was used when heterogeneity was ≤25%. A two-side alpha value less than 0.05 was considered statistically significant. Publication bias was assessed using the funnel plot asymmetry. All analyses were performed using the OpenMeta (Analyst) [52].

## Results

### Study characteristics and quality assessment

**Fig. 2:**
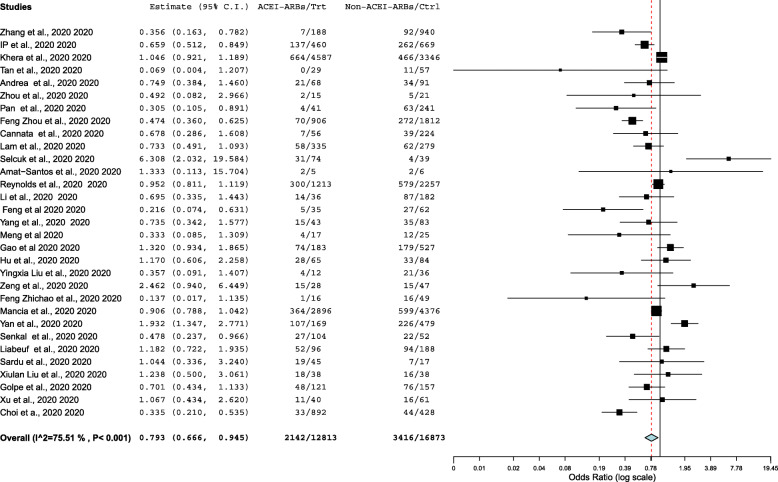
The risk of poor COVID-19 clinical outcome with ACEI/ARBs compared to Non-ACEI/ARBs

A total of 1442 potentially relevant articles were identified through our search strategy. Of these, 36 articles were included in our final analysis (Fig. 2). All the included articles were of good quality (NOS score ≥ 7), and study characteristics and quality assessment are shown in Table 1 and Table S2, respectively.

A total of 30,795 COVID-19 patients were included. Among these, 19.6% (6036/30,795) of them had poor COVID-19 outcome. Majority of these patients (55% or 16,873/30,795) were taking non-RAAS inhibitors, whereas 45% (13,922/30,795) were receiving RAAS inhibitors. In most of the studies (22 of the 36 studies) patients taking antihypertensives were categorized based on the severity of COVID-19, whereas in the remaining 14 studies they were categorized based on survival after COVID-19 (Table 1). Eighteen studies compared RAAS inhibitors to non-RAAS inhibitors without mentioning of a specific antihypertensive sub-class whereas the remaining 18 studies documented the number of patients taking a specific drug class within the RAAS inhibitor and non-RAAS inhibitor drug groups. The latter group of studies that documented specific drug classes were eligible for sub-meta-analyses. In these studies, the total number of patients taking ACEIs (5145) and ARBs (5250) were comparable. In addition, the number of patients taking CCBs (3102), BBs (2792), and thiazides (1797) were approximately comparable (Table 1).

### Comparison of the risk of poor COVID-19 clinical outcomes with different antihypertensives

We found that the overall risk of poor patient outcomes was lower in patients taking RAAS inhibitors (OR = 0.79, 95% CI: [0.67, 0.95]) compared with those taking non-RAAS inhibitors (Fig. 2). Specific comparison of ACEIs to different antihypertensives including ARBs, CCBs, thiazides did not bring a decrease in poor outcomes among COVID-19 patients (Table 2, Supplementary Figures S1-S13). Similarly, comparison of ARBs to these class of drugs did not show a significant improvement in outcomes. For example, it is interesting to note that a comparison of ARBs to CCBs (OR = 0.90, 95% CI: [0.62, 1.33]) did not show difference in poor COVID-19 outcomes. However, comparison of either ACEIs or ARBs to BBs showed a decrease in poor COVID-19 outcomes (OR = 0.85, 95% CI: [0.73, 0.99]) and (OR = 0.72, 95% CI: [0.55, 0.94]), respectively.

**Table 2:** Risk of poor COVID-19 clinical outcomes with different classes of antihypertensives

Comparision	Odds ratio (meta-analysis)	95% CI	Method of analysis	Number of studies included in the sub-meta-analysis	Forest plot
ACEI to ARBs	0.94	0.84–1.04	MH	16	Figure S1
ACEIs to BBs	0.85	0.73–0.99	MH	7	Figure S2
ACEIs to CCBs	0.91	0.67–1.23	RE	8	Figure S3
ACEIs to Thiazides	1.22	1.02–1.45	MH	6	Figure S4
ACEIs to all other antihypertensives	0.91	0.84–0.99	MH	16	Figure S5
ARBs to all other antihypertensives	0.98	0.83–1.17	RE	16	Figure S6
ARBs to BBs	0.72	0.55–0.94	RE	7	Figure S7
ARBs to CCBs	0.90	0.62–1.33	RE	8	Figure S8
ARBs to Thiazides	1.15	0.97–1.37	MH	6	Figure S9
ARBs to all other non-RAAS antihypertensives	0.89	0.71–1.12	RE	11	Figure S10
ACEIs to all other non-RAAS antihypertensives	0.89	0.74–1.06	RE	11	Figure S11
CCBs to ACEI, ARBs, BBs	0.95	0.68–1.33	RE	10	Figure S12
ACEI, ARBs, BBs to CCBs and thiazides	1.13	0.87–1.47	RE	10	Figure S13

*Abbreviations*: *ACEI* angiotensin-I-converting enzyme inhibitors, *ARBs* angiotensin II receptor blockers, *BBs* Beta blockers, *CCBs* calcium channel blockers, *MH* Mantel-Haenszel, *RE* random-effects. Figures S1-S13 are found in the supplementary file

## Discussion

Evidence on the safety of antihypertensive medications is of paramount importance as about one-third of the world’s population is estimated to have hypertension [53] and this comorbidity is associated with increased mortality in patients with COVID-19 [54]. Since RAAS inhibitors were reported to affect the clinical outcome of COVID-19, either for good or worse [6, 11, 55], we pooled recent studies to provide stronger evidence on the effects of these drugs. In addition, we also performed multiple sub-meta-analyses (comparing class of antihypertensives) to identify the effect of specific drug classes. We found that COVID-19 patients taking RAAS inhibitors had an overall decreased risk of poor outcomes compared to those receiving non-RAAS inhibitors. However, based on our multiple sub-meta-analysis findings (Table 2), these effects were likely related to the underlying comorbidities for which specific antihypertensive class of drugs were indicated, and not necessarily related to the beneficiary role of RAAS inhibitors. In addition to compelling comorbidity, the adverse effects of drugs such as BBs could also be responsible.

It is possible that the overall decreased risk of COVID-19 severity or mortality with the use of RAAS inhibitors could be related to the blockage of a rapidly progressing systemic inflammation that is frequently seen in severe COVID-19 cases [56]. For example, COVID-19 patients taking ACE/ARBs had lower levels of inflammatory markers, such as interleukin 6 (IL-6) [9], C-reactive protein (CRP) and procalcitonin [10], than those not taking these drugs. In addition, these classes of drugs could also help prevent hypokalaemia, a complication that was reported to occur in COVID-19 patients [57]. Hence, RAAS inhibitors may decrease poor clinical outcomes by limiting the deleterious effects of angiotensin-II in multisystem inflammation, as well as by preventing the occurrence of hypokalaemia [56, 57]. Further, these drugs could also circumvent SARS-CoV-2 induced ACE2 downregulation in host cells, so that the preventive effects of ACE2 against severe disease are not lost [58].

However, the apparent decrease in COVID-19 poor outcomes with RAAS inhibitors could also be due to the mere comorbidity differences among patients who took different class of antihypertensive drugs. This is supported by our sub-meta-analyses findings that showed both ACEIs and ARBs were not different from CCBs in terms of COVID-19 outcomes (Table 2). Interestingly, however, ACEIs and ARBs showed a decrease in poor COVID-19 outcomes, when each were compared to BBs (Table 2). Therefore, the overall decrease in poor COVID-19 outcomes with RAAS inhibitors relative to non-RAAS inhibitors could be related to more severe cardiovascular comorbidity in patients taking certain non-RAAS inhibitors like BBs. Further, some adverse effects of BBs could be the cause of poor COVID-19 clinical outcomes.

In fact, a recent study showed that the use of either ACEIs or ARBs does not increase ACE2 expression in human tissues [59]. This is in sharp contrast to a previous experimental study (in rats) that reported an increase in ACE2 expression with these drugs [3]. Note that, increased ACE2 expression with the use of RAAS inhibitors was the key pathophysiologic process that was hypothesised to be associated with an increase in SARS-CoV-2 entry to human cells and hence diseases severity. On the other hand, increased ACE2 expression was also thought to be associated with a decrease in COVID-19 severity and mortality, since ACE2 enhances the degradation of harmful angiotensins into cardioprotective ones. Hence, combining all the above evidences, RAAS inhibitor antihypertensive medications might not have any effect at all on the severity or mortality of COVID-19.

To the best of our knowledge, this systematic review and meta-analysis is a comprehensive one including the most recent studies and clinical outcomes of COVID-19 among patients taking major classes of antihypertensive drugs. However, our study has some limitations, majority of which are implicit to the studies included. First, even though all of the included papers were of good quality, propensity matching to address common confounders (e.g., age, comorbidity) was performed in only few of the studies. Second, the number of studies included in our sub-meta-analyses (versus the main meta-analysis) (Table 2) were relatively small and this might affect our conclusions. The other limitation is that our interpretation of sub-meta-analysis findings were based on our clinical judgement that assumed prescription of BBs could occur in patients with worse cardiovascular comorbidity [16]. For instance, patients taking certain antihypertensives like BBs may not necessarily have a worse cardiovascular condition. Similarly, even though ACEIs are good choice of antihypertensives in patients without any comorbidity, they are also preferred drugs in those who had myocardial infarction or systolic dysfunction. Finally, this review was not able to measure the clinical outcome of COVID-19 patients taking the combination of RAAS inhibitor and non-RAAS inhibitor drugs.

On the other hand, the strength of this meta-analysis is that we excluded studies that compared hypertensive patients who were taking RAAS inhibitors to those that were not taking any form of antihypertensive (e.g., on dietary management). This helped us to have comparable groups in terms of comorbidity and severity of hypertension.

## Conclusion

An increased risk of severe COVID-19 or death was unlikely in patients receiving RAAS inhibitors (Fig. 2). Differences in COVID-19 poor outcomes were likely due to the underlying comorbidities for which the antihypertensive drugs were prescribed. COVID-19 should not bring a discontinuation or change in treatment with RAAS inhibitors as these antihypertensive drugs might not have any effect at all on the disease severity or mortality of COVID-19.

## Supplementary Information

**Additional file 1: Table S1.** PRISMA Checklist. **Table S2.** Quality score of articles (Newcastle–Ottawa Scale). **Figure S1.** Risk of poor COVID-19 clinical outcome with ACEIs relative to ARBs. **Figure S2.** Risk of poor COVID-19 clinical outcome with ACEIs relative to BBs. **Figure S3.** Risk of poor COVID-19 clinical outcome with ACEIs relative to CCBs. **Figure S4.** Risk of poor COVID-19 clinical outcome with ACEIs relative to thiazides. **Figure S5.** Risk of poor COVID-19 clinical outcome with ACEIs relative to all other antihypertensives. **Figure S6.** Risk of poor COVID-19 clinical outcome with ARBs relative to all other antihypertensives. **Figure S7.** Risk of poor COVID-19 clinical outcome with ARBs relative to BBs. **Figure S8.** Risk of poor COVID-19 clinical outcome with ARBs relative to CCBs. **Figure S9.** Risk of poor COVID-19 clinical outcome with ARBs relative to thiazides. **Figure S10.** Risk of poor COVID-19 clinical outcome with ARBs relative to all other non-RAAS antihypertensives. **Figure S11.** Risk of poor COVID-19 clinical outcome with ACEIs relative to all other non-RAAS antihypertensives. **Figure S12.** Risk of poor COVID-19 clinical outcome with CCBs relative to ACEI, ARBs, BBs. **Figure S13.** Risk of poor COVID-19 clinical outcome with ACEI, ARBs, BBs relative to CCBs and thiazides.

## Data Availability

The datasets supporting the conclusions of this article are included within the article and its additional file. Supplementary Tables S1-S2 and Supplementary Figures S1-S13 are found in the supplementary file.

## Abbreviations

ACE2: Angiotensin-converting enzyme 2
ACEI: Angiotensin-I-converting enzyme inhibitors
ARBs: Angiotensin II receptor blockers
BBs: Beta blockers
CCBs: Calcium channel blockers
COVID-19: Coronavirus disease-19
CRP: C-reactive protein
CVD: Cardiovascular diseases
FiO2: Percentage of inspired oxygen
HTN: Hypertension
IL-6: Interleukin 6
mm Hg: Millimetre of mercury
NOS: Newcastle-Ottawa quality assessment scale
OR: Odds ratio
PaO2: Partial pressure of arterial oxygen
RAAS: Renin–angiotensin–aldosterone system
SARS-CoV-2: Severe acute respiratory syndrome coronavirus 2
