# Supplementary material for: Comparison of renin–angiotensin–aldosterone system inhibitors with other antihypertensives in association with coronavirus disease-19 clinical outcomes
Source: BMC Infect Dis. 2021 Jun 5;21:527. doi: 10.1186/s12879-021-06088-6 (PMC8178664; doi:10.1186/s12879-021-06088-6)
Supplement: Supplementary file 1 — Additional file 1: Table S1. PRISMA Checklist. Table S2. Quality score of articles (Newcastle–Ottawa Scale). Figure S1. Risk of poor COVID-19 clinical outcome with ACEIs relative to ARBs. Figure S2. Risk of poor COVID-19 clinical outcome with ACEIs relative to BBs. Figure S3. Risk of poor COVID-19 clinical outcome with ACEIs relative to CCBs. Figure S4. Risk of poor COVID-19 clinical outcome with ACEIs relative to thiazides. Figure S5. Risk of poor COVID-19 clinical outcome with ACEIs relative to all other antihypertensives. Figure S6. Risk of poor COVID-19 clinical outcome with ARBs relative to all other antihypertensives. Figure S7. Risk of poor COVID-19 clinical outcome with ARBs relative to BBs. Figure S8. Risk of poor COVID-19 clinical outcome with ARBs relative to CCBs. Figure S9. Risk of poor COVID-19 clinical outcome with ARBs relative to thiazides. Figure S10. Risk of poor COVID-19 clinical outcome with ARBs relative to all other non-RAAS antihypertensives. Figure S11. Risk of poor COVID-19 clinical outcome with ACEIs relative to all other non-RAAS antihypertensives. Figure S12. Risk of poor COVID-19 clinical outcome with CCBs relative to ACEI, ARBs, BBs. Figure S13. Risk of poor COVID-19 clinical outcome with ACEI, ARBs, BBs relative to CCBs and thiazides. [file 12879_2021_6088_MOESM1_ESM.docx]

**Appendices**

**Table S1: PRISMA Checklist**

| **Section/topic** | **#** | **Checklist item** | | **Reported on page #** |
| --- | --- | --- | --- | --- |
| **TITLE** | | | |  |
| Title | 1 | Identify the report as a systematic review, meta-analysis, or both. | | p 1 |
| **ABSTRACT** | | | |  |
| Structured summary | 2 | Provide a structured summary including, as applicable: background; objectives; data sources; study eligibility criteria, participants, and interventions; study appraisal and synthesis methods; results; limitations; conclusions and implications of key findings; systematic review registration number. | | p 2 |
| **INTRODUCTION** | | | |  |
| Rationale | 3 | Describe the rationale for the review in the context of what is already known. | | p 3 |
| Objectives | 4 | Provide an explicit statement of questions being addressed with reference to participants, interventions, comparisons, outcomes, and study design (PICOS). | | p 3 |
| **METHODS** | | | |  |
| Protocol and registration | 5 | Indicate if a review protocol exists, if and where it can be accessed (e.g., Web address), and, if available, provide registration information including registration number. | | _ |
| Eligibility criteria | 6 | Specify study characteristics (e.g., PICOS, length of follow-up) and report characteristics (e.g., years considered, language, publication status) used as criteria for eligibility, giving rationale. | | p.5 |
| Information sources | 7 | Describe all information sources (e.g., databases with dates of coverage, contact with study authors to identify additional studies) in the search and date last searched. | | p.4 |
| Search | 8 | Present full electronic search strategy for at least one database, including any limits used, such that it could be repeated. | | pp.4-5 |
| Study selection | 9 | State the process for selecting studies (i.e., screening, eligibility, included in systematic review, and, if applicable, included in the meta-analysis). | | p. 4-5 |
| Data collection process | 10 | Describe method of data extraction from reports (e.g., piloted forms, independently, in duplicate) and any processes for obtaining and confirming data from investigators. | | p 4 |
| Data items | 11 | List and define all variables for which data were sought (e.g., PICOS, funding sources) and any assumptions and simplifications made. | | p 4 |
| Risk of bias in individual studies | 12 | | Describe methods used for assessing risk of bias of individual studies (including specification of whether this was done at the study or outcome level), and how this information is to be used in any data synthesis. | p 6 |
| Summary measures | 13 | | State the principal summary measures (e.g., risk ratio, difference in means). | pp 4-6 |
| Synthesis of results | 14 | | Describe the methods of handling data and combining results of studies, if done, including measures of consistency (e.g., I^2^) for each meta-analysis. | pp 5-6 |

| Risk of bias across studies | | 15 | Specify any assessment of risk of bias that may affect the cumulative evidence (e.g., publication bias, selective reporting within studies). | p 6 |  |
| --- | --- | --- | --- | --- | --- |
| **Section/topic** | | **#** | | **Checklist item** | **Reported on page #** |
| Additional analyses | | 16 | Describe methods of additional analyses (e.g., sensitivity or subgroup analyses, meta-regression), if done, indicating which were pre-specified. |  |  |
| **RESULTS** | | | |  |  |
| Study selection | | 17 | Give numbers of studies screened, assessed for eligibility, and included in the review, with reasons for exclusions at each stage, ideally with a flow diagram. | p 6, Flow diagram (Figure) 1 |  |
| Study characteristics | | 18 | For each study, present characteristics for which data were extracted (e.g., study size, PICOS, follow-up period) and provide the citations. | pp 5-6  Supplementary Table 2 |  |
| Risk of bias within studies | | 19 | Present data on risk of bias of each study and, if available, any outcome level assessment (see item 12). |  |  |
| Results of individual studies | | 20 | For all outcomes considered (benefits or harms), present, for each study: (a) simple summary data for each intervention group (b) effect estimates and confidence intervals, ideally with a forest plot. | pp 6-7 Tables 1 and 2 |  |
| Synthesis of results | | 21 | Present results of each meta-analysis done, including confidence intervals and measures of consistency. | pp 6-7 Table 2, Figure 2, Supplementary Figures S1 - S10 |  |
| Risk of bias across studies | | 22 | Present results of any assessment of risk of bias across studies (see Item 15). | pp 6 |  |
| Additional analysis | | 23 | Give results of additional analyses, if done (e.g., sensitivity or subgroup analyses, meta-regression [see Item 16]). |  |  |
| **DISCUSSION** | | | |  |  |
| Summary of evidence | | 24 | Summarize the main findings including the strength of evidence for each main outcome; consider their relevance to key groups (e.g., healthcare providers, users, and policy makers). | pp 8-9 |  |
| Limitations | | 25 | Discuss limitations at study and outcome level (e.g., risk of bias), and at review-level (e.g., incomplete retrieval of identified research, reporting bias). | p 9 |  |
| Conclusions | | 26 | Provide a general interpretation of the results in the context of other evidence, and implications for future research. | p 10 |  |
| **FUNDING** | | | |  |  |
| Funding | | 27 | Describe sources of funding for the systematic review and other support (e.g., supply of data); role of funders for the systematic review. | 10 |  |
|  | | | | |  |

**Table S2: Quality score of articles (Newcastle–Ottawa Scale)**

|  |  | Selection (representativeness of exposed cohort, selection of the non-exposed cohort, ascertainment of exposure, at the start of the study the outcome of interest was not present) | | | | Comparability (study design and analysis, and whether any confounding variables were adjusted) | Outcome (follow-up period, cohort retention, ascertained by independent blind assessment, record linkage, or self-report) | | |  |
| --- | --- | --- | --- | --- | --- | --- | --- | --- | --- | --- |
| Serial number | Study | Representativeness of Exposed Cohort (max: **) | Selection of the Non-Exposed Cohort from Same Source as Exposed Cohort: (*) | Ascertainment of Exposure (**) | Outcome of Interest Was Not Present at Start of Study (yes=*) | Comparability of Cohorts (**) | Assessment Outcome (**) | Follow-Up Long Enough for Outcome to Occur (*) | Adequacy of Follow-Up (**) | Quality Score |
| 1 | Zhang et al., 2020 | Yes ★ ★ | Yes ★ | ★ | Yes ★ | Yes ★ ★ | ★ | ★ | ★ | Good |
| 2 | IP et al., 2020 | ★ | Yes ★ | Yes ★ | NO | Yes ★ | ★ | ★ | ★ | Good |
| 3 | Khera et al., 2020 | ★ | Yes ★ | Yes ★ | NO | Yes ★ |  |  |  | Good |
| 4 | Richardson et al., 2020 | ★ | Yes ★ | Yes ★ | NO | Yes ★ | ★ | ★ | ★ | Good |
| 5 | Tan et al., 2020 | ★ | Yes ★ | Yes ★ | NO | Yes ★ | ★ | ★ | ★ | Good |
| 6 | Andrea et al., 2020 | ★ | Yes ★ | Yes ★ | NO | Yes ★ | ★ | ★ | ★ | Good |
| 7 | Xian Zhou et al., 2020 | ★ | Yes ★ | Yes ★ | NO | Yes ★ | ★ | ★ | ★ | Good |
| 8 | Feng Zhou et al., 2020 | ★ | Yes ★ | Yes ★ | NO | Yes ★ | ★ | ★ | ★ | Good |
| 9 | Pan et al., 2020 | ★ | Yes ★ | Yes ★ | NO | Yes ★ | ★ | ★ | ★ | Good |
| 10 | Cannata et al., 2020 | ★ | Yes ★ | Yes ★ | Yes ★ | Yes ★ | ★ | ★ | ★ | Good |
| 11 | Lam et al., 2020 | ★ | Yes ★ | Yes ★ | Yes ★ | Yes ★ ★ | ★ | ★ | ★ | Good |
| 12 | Selcuk et al., 2020 | ★ | Yes ★ | Yes ★ | NO | Yes ★ | ★ | ★ | ★ | Good |
| 13 | Amat-Santos et al., 2020 | ★ | Yes ★ | Yes ★ | NO | Yes ★ | ★ | ★ | ★ | Good |
| 14 | Felice et al., 2020 | ★ | Yes ★ | Yes ★ | Yes ★ | Yes ★ | ★ | ★ | ★ | Good |
| 15 | Reynolds et al., 2020 | ★★ | Yes ★ | Yes ★ | No | Yes ★ ★ | ★ | ★ | ★ | Good |
| 16 | Li et al., 2020 | ★ | Yes ★ | Yes ★ | NO | Yes ★ | ★ | ★ | ★ | Good |
| 17 | Feng et al., 2020 | ★ | Yes ★ | Yes ★ | Yes ★ | Yes ★ | ★ | ★ | ★ | Good |
| 18 | Yang et al., 2020 | ★ | Yes ★ | Yes ★ | No | Yes ★ | ★ | ★ | ★ | Good |
| 19 | Meng et al., 2020 | ★ | Yes ★ | Yes ★ | No | Yes ★ | ★ | ★ | ★ | Good |
| 20 | Gao et al., 2020 | ★ | Yes ★ | Yes ★ | NO | Yes ★ | ★ | ★ | ★ | Good |
| 21 | Hu et al., 2020 | ★ | Yes ★ | Yes ★ | NO | Yes ★ | ★ | ★ | ★ | Good |
| 22 | Liu et al., 2020 | ★ | Yes ★ | Yes ★ | NO | Yes ★ | ★ | ★ | ★ | Good |
| 23 | Zeng et al., 2020 | ★ | Yes ★ | Yes ★ | NO | Yes ★ | ★ | ★ | ★ | Good |
| 24 | Bravi et al., 2020 | NO | Yes ★ | Yes ★ | NO | Yes ★ | ★ | ★ | ★ | fair |
| 25 | Dauchet et al., 2020 | NO | Yes ★ | Yes ★ | NO | Yes ★ | ★ | ★ | ★ | fair |
| 26 | Feng Zhichao et al., 2020 | ★ | Yes ★ | Yes ★ | NO | Yes ★ | ★ | ★ | ★ | Good |
| 27 | Mancia et al., 2020 | ★ | Yes ★ | Yes ★ | NO | Yes ★ | ★ | ★ | ★ | Good |
| 28 | Yan et al., 2020 | ★ | Yes ★ | Yes ★ | NO | Yes ★ | ★ | ★ | ★ | Good |
| 29 | Senkal et al., 2020 | ★ | Yes ★ | Yes ★ | NO | Yes ★ | ★ | ★ | ★ | Good |
| 30 | Liabeuf et al., 2020 | ★ | Yes ★ | Yes ★ | NO | Yes ★ | ★ | ★ | ★ | Good |
| 31 | Sardu et al., 2020 | ★ | Yes ★ | Yes ★ | Yes ★ | Yes ★ | ★ | ★ | ★ | Good |
| 32 | Xiulan Liu et al., 2020 | ★ | Yes ★ | Yes ★ | NO | Yes ★ | ★ | ★ | ★ | Good |
| 33 | Lopez-Otero et al., 2020 | ★ | Yes ★ | Yes ★ | NO | Yes ★ | ★ | ★ | ★ | Good |
| 34 | Golpe et al., 2020 | ★ | Yes ★ | Yes ★ | NO | Yes ★ | ★ | ★ | ★ | Good |
| 35 | Xu et al., 2020 | ★ | Yes ★ | Yes ★ | NO | Yes ★ | ★ | ★ | ★ | Good |
| 36 | Choi et al., 2020 | ★ | Yes ★ | Yes ★ | NO | Yes ★ | ★ | ★ | ★ | Good |

Interpretation: Good quality: 3 or 4 stars (★) in selection domain AND 1 or 2 stars in comparability domain AND 2 or 3 stars in outcome domain; Fair quality: 2 stars in selection domain AND 1 or 2 stars in comparability domain AND 2 or 3 stars in outcome/exposure domain; Poor quality: 0 or 1 star in selection domain OR 0 stars in comparability domain OR 0 or 1 stars in outcome/exposure domain.


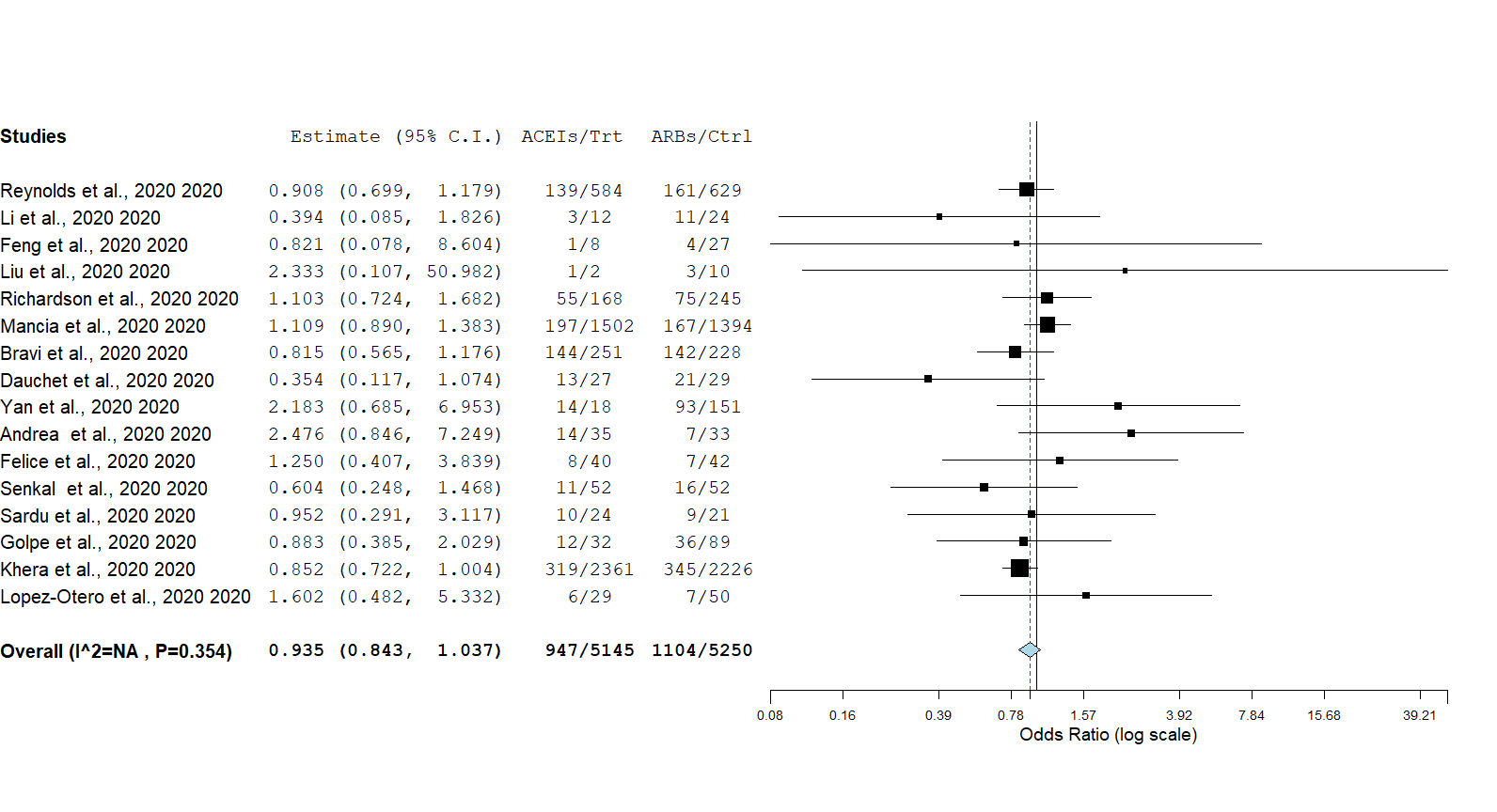


Figure S1: Risk of poor COVID-19 clinical outcome with ACEIs relative to ARBs.


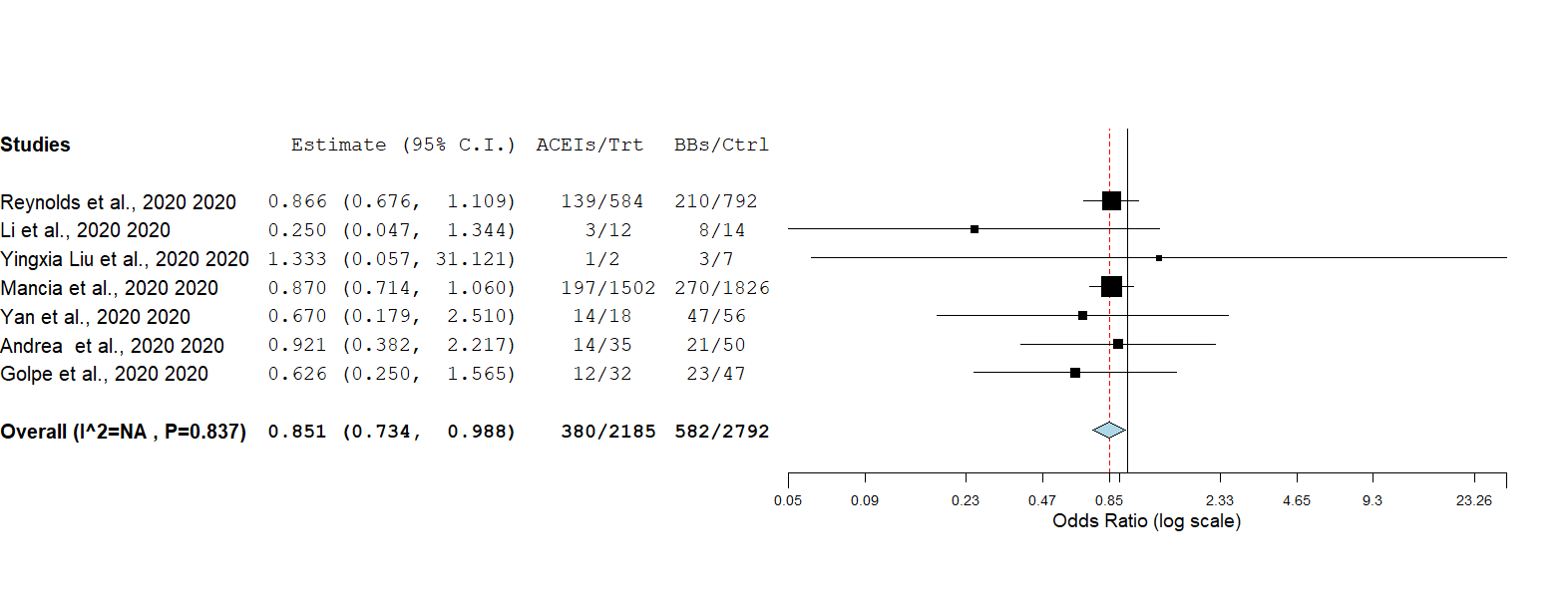


Figure S2: Risk of poor COVID-19 clinical outcome with ACEIs relative to BBs.


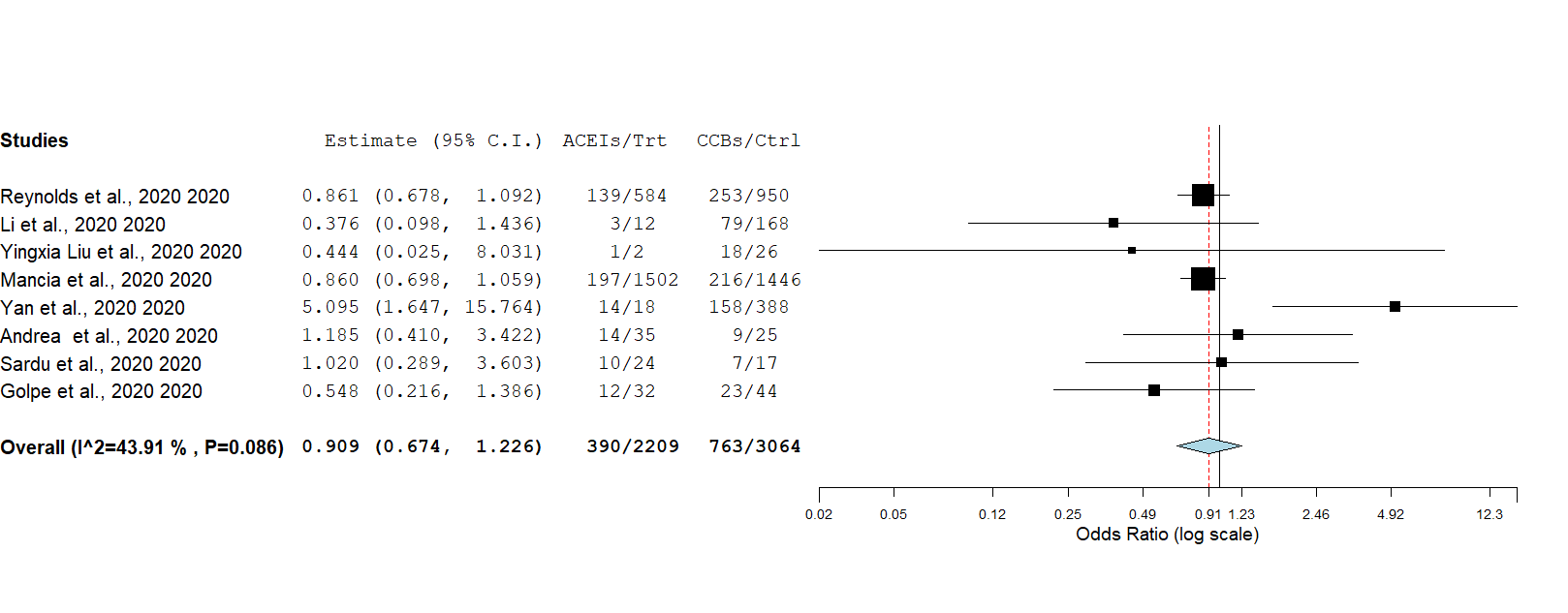


Figure S3: Risk of poor COVID-19 clinical outcome with ACEIs relative to CCBs.


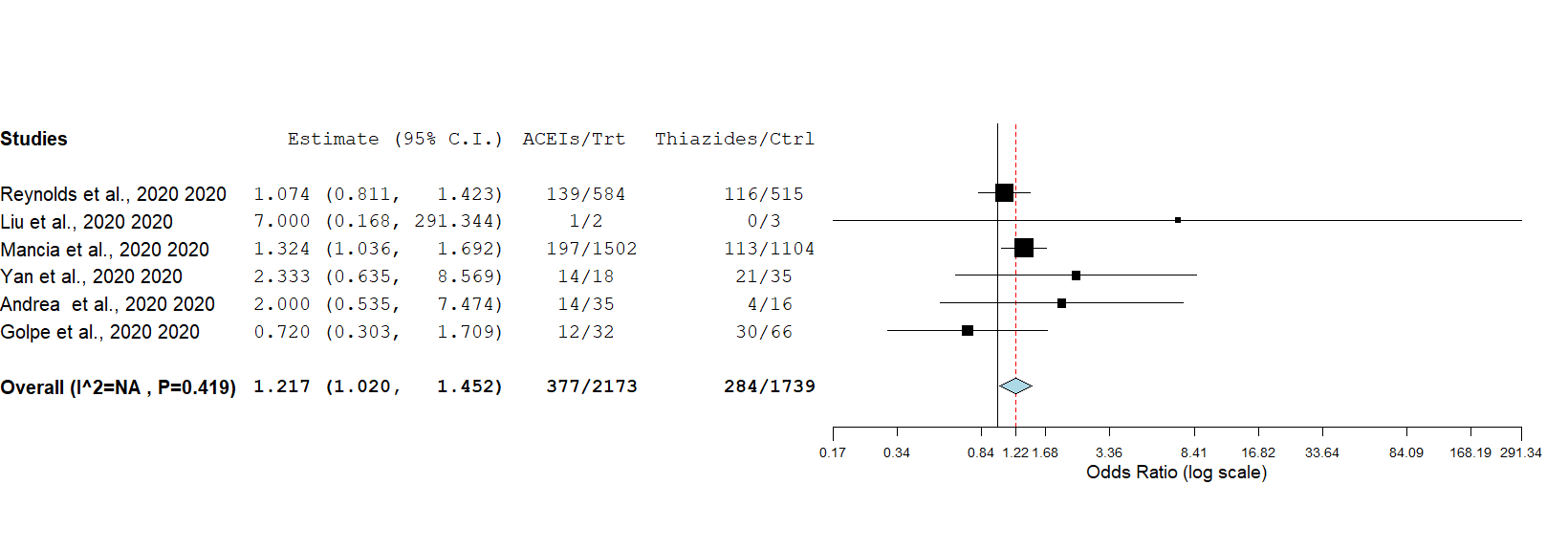


Figure S4: Risk of poor COVID-19 clinical outcome with ACEIs relative to thiazides.


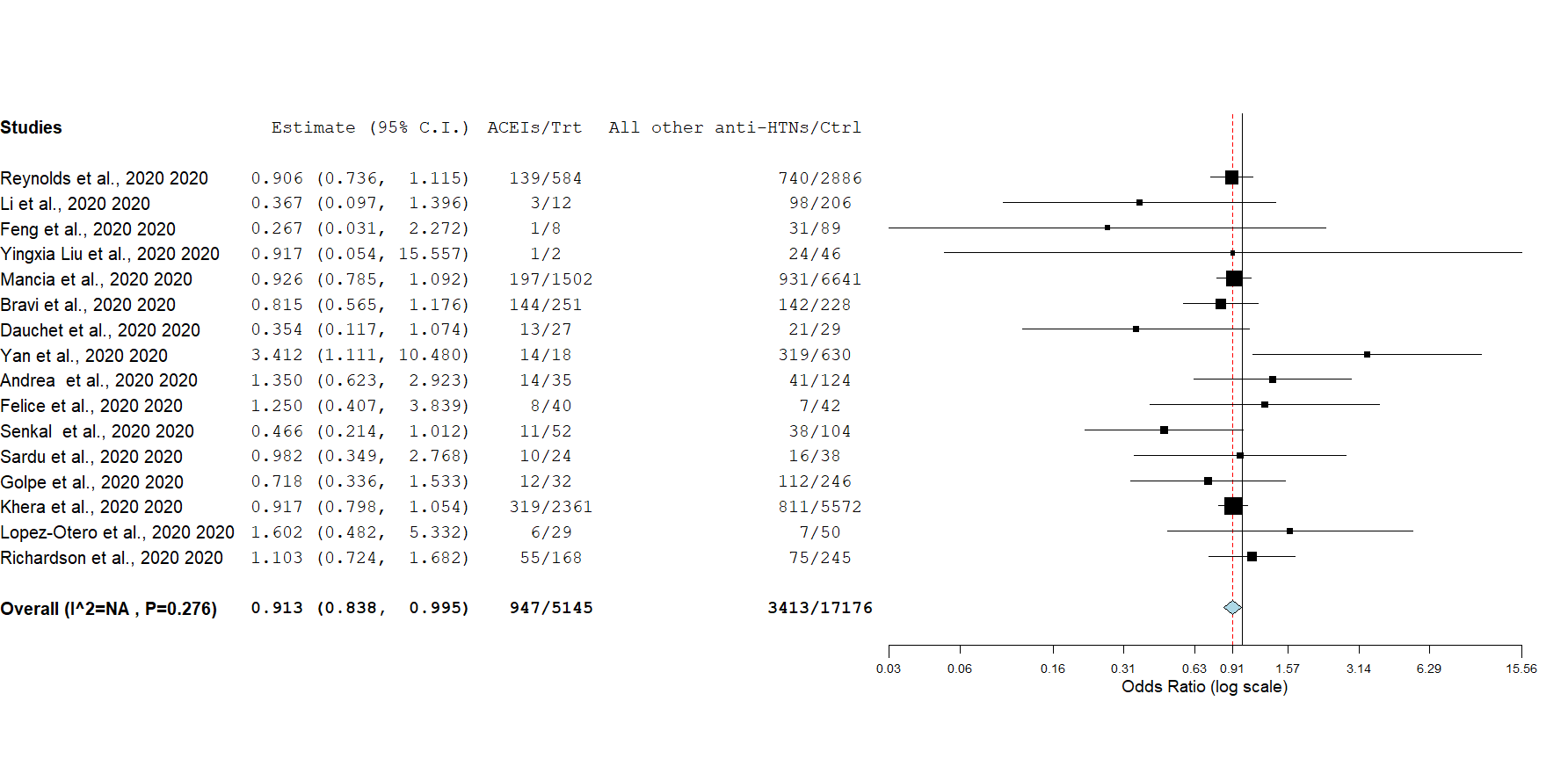


Figure S5: Risk of poor COVID-19 clinical outcome with ACEIs relative to all other antihypertensives.


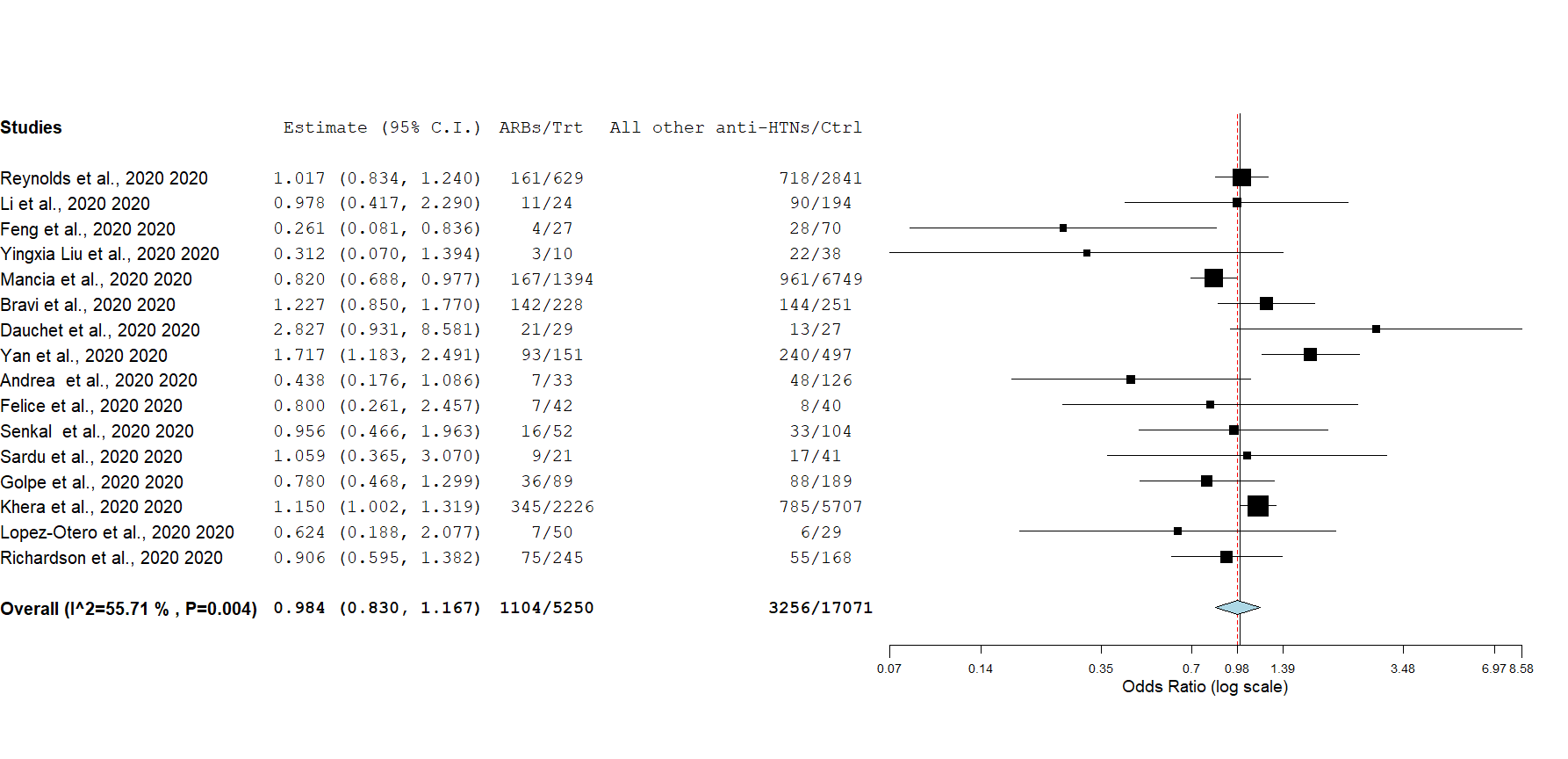


Figure S6: Risk of poor COVID-19 clinical outcome with ARBs relative to all other antihypertensives


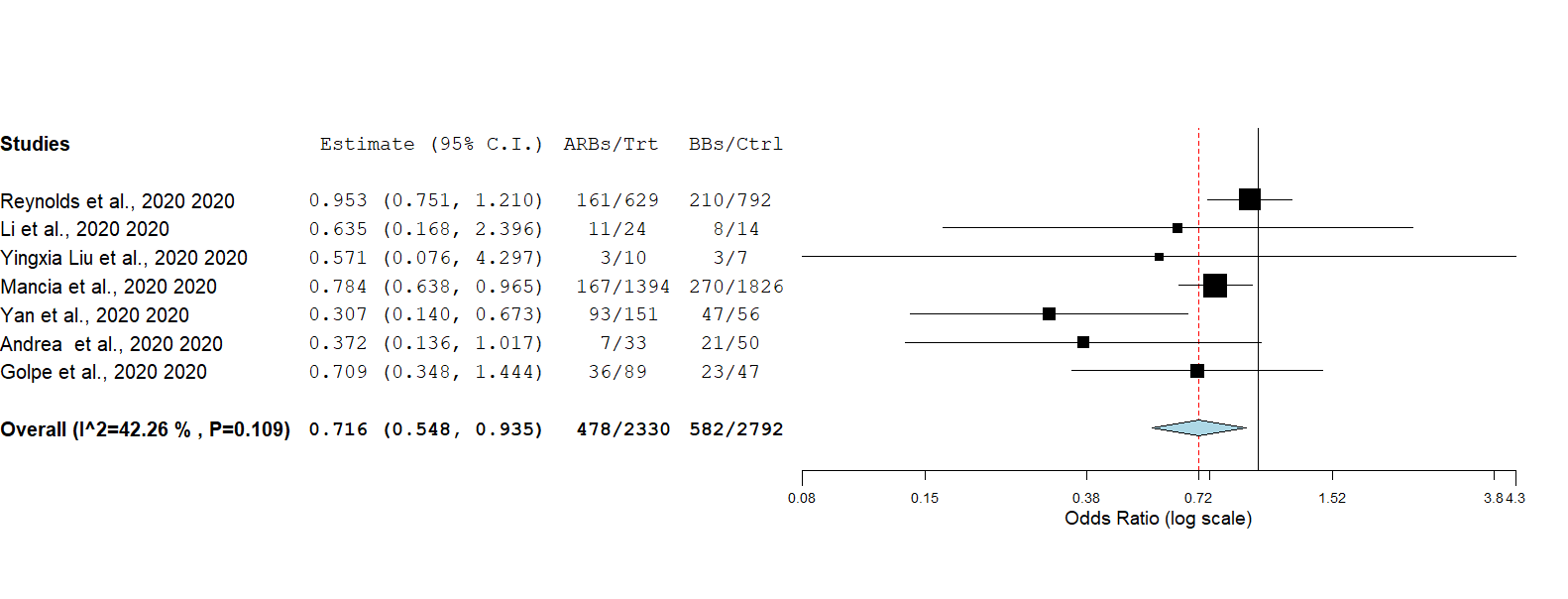


Figure S7: Risk of poor COVID-19 clinical outcome with ARBs relative to BBs.


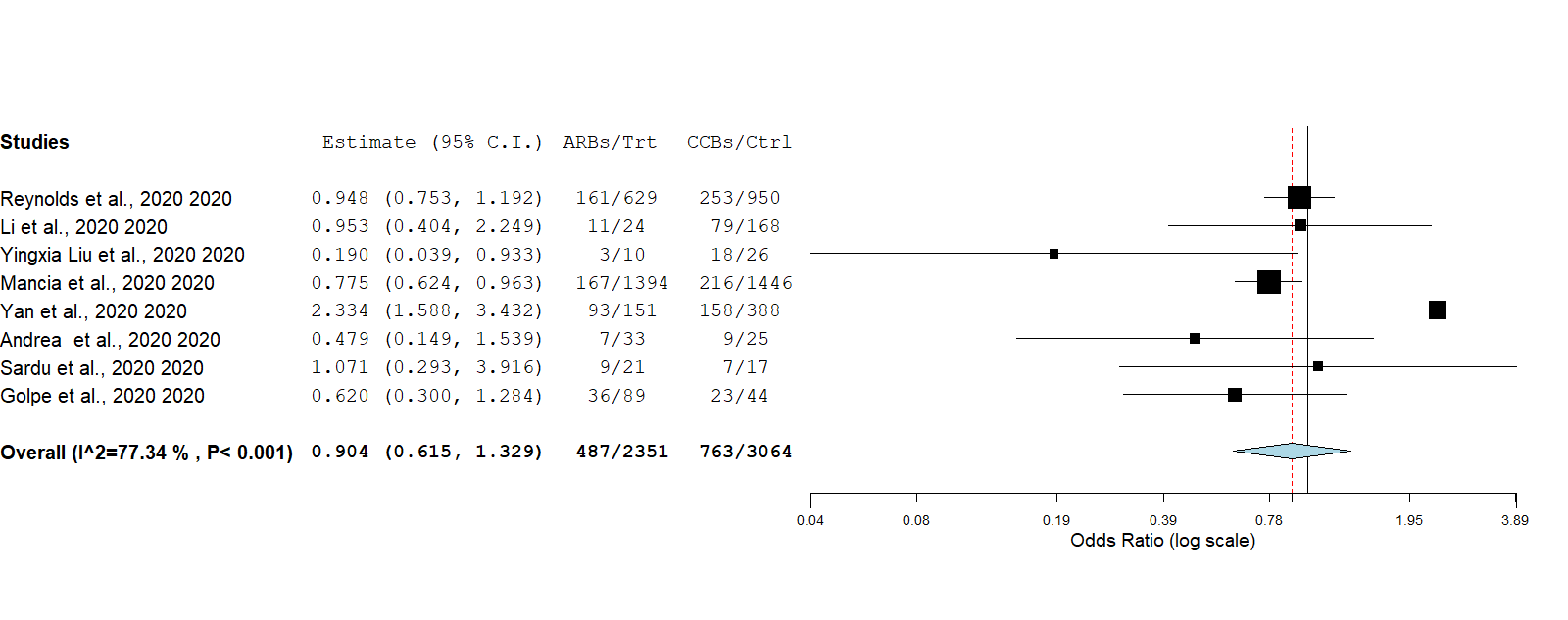


Figure S8: Risk of poor COVID-19 clinical outcome with ARBs relative to CCBs.


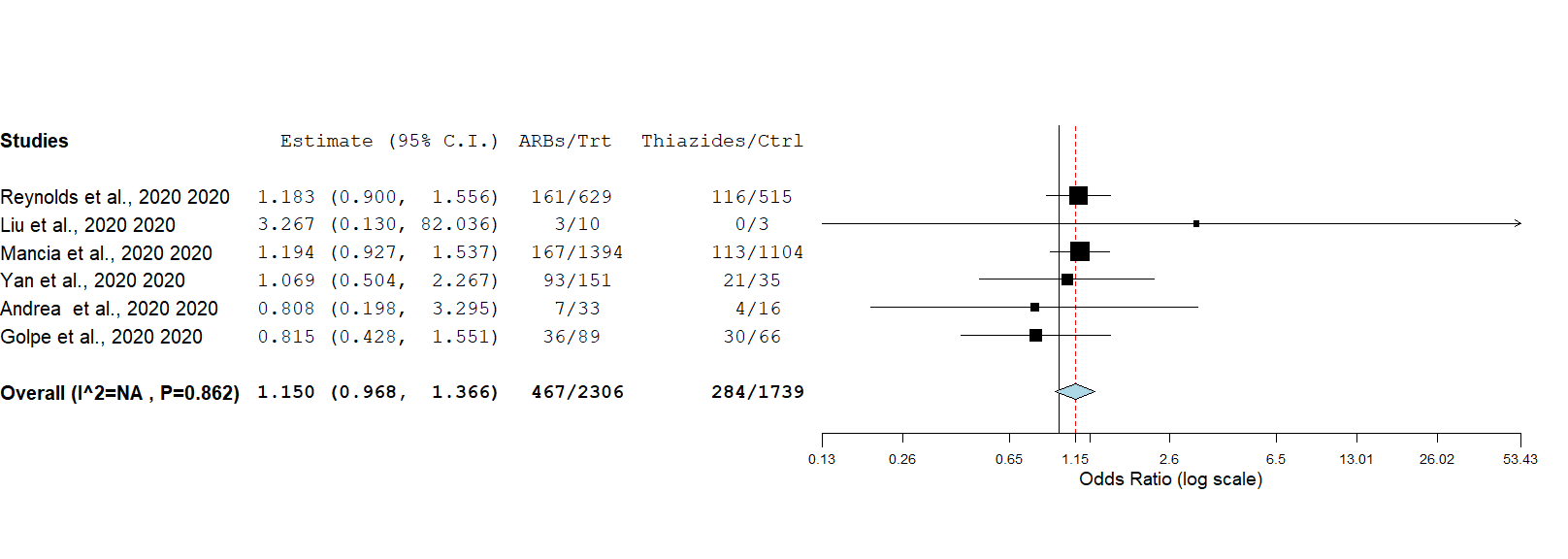


Figure S9: Risk of poor COVID-19 clinical outcome with ARBs relative to thiazides.

.


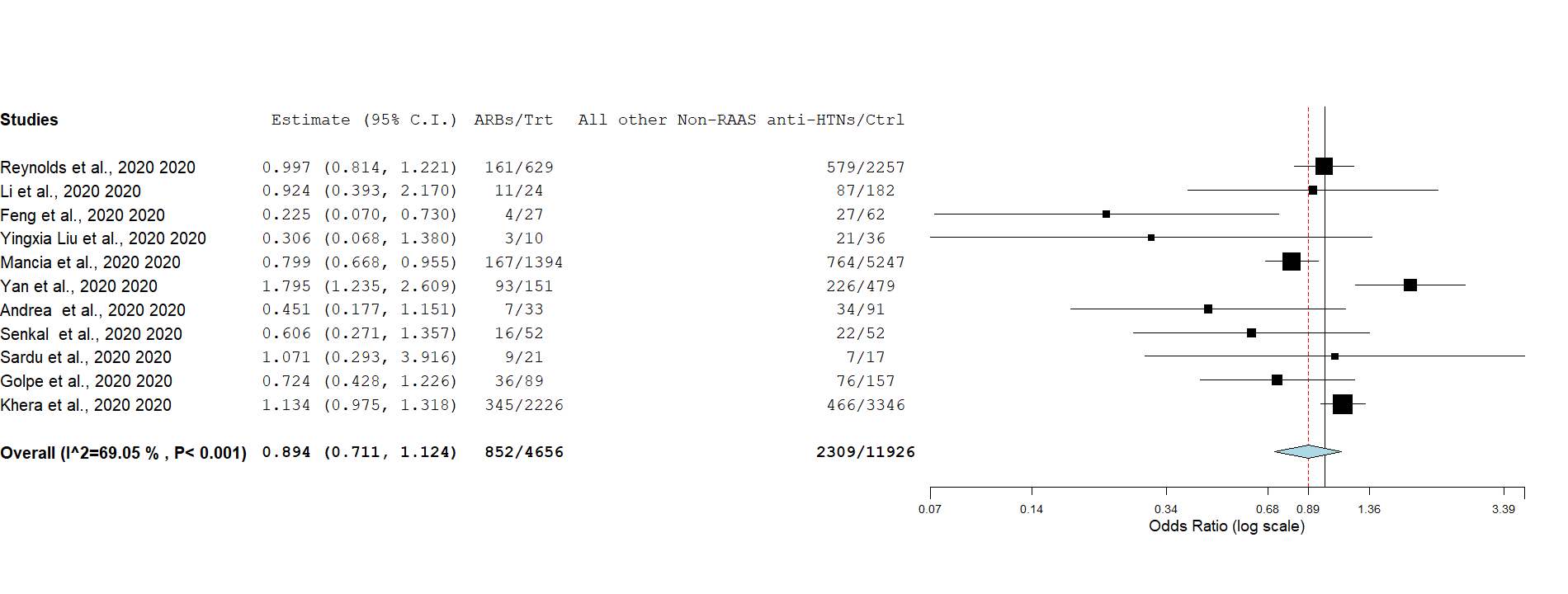


Figure S10: Risk of poor COVID-19 clinical outcome with ARBs relative to all other non-RAAS antihypertensives.


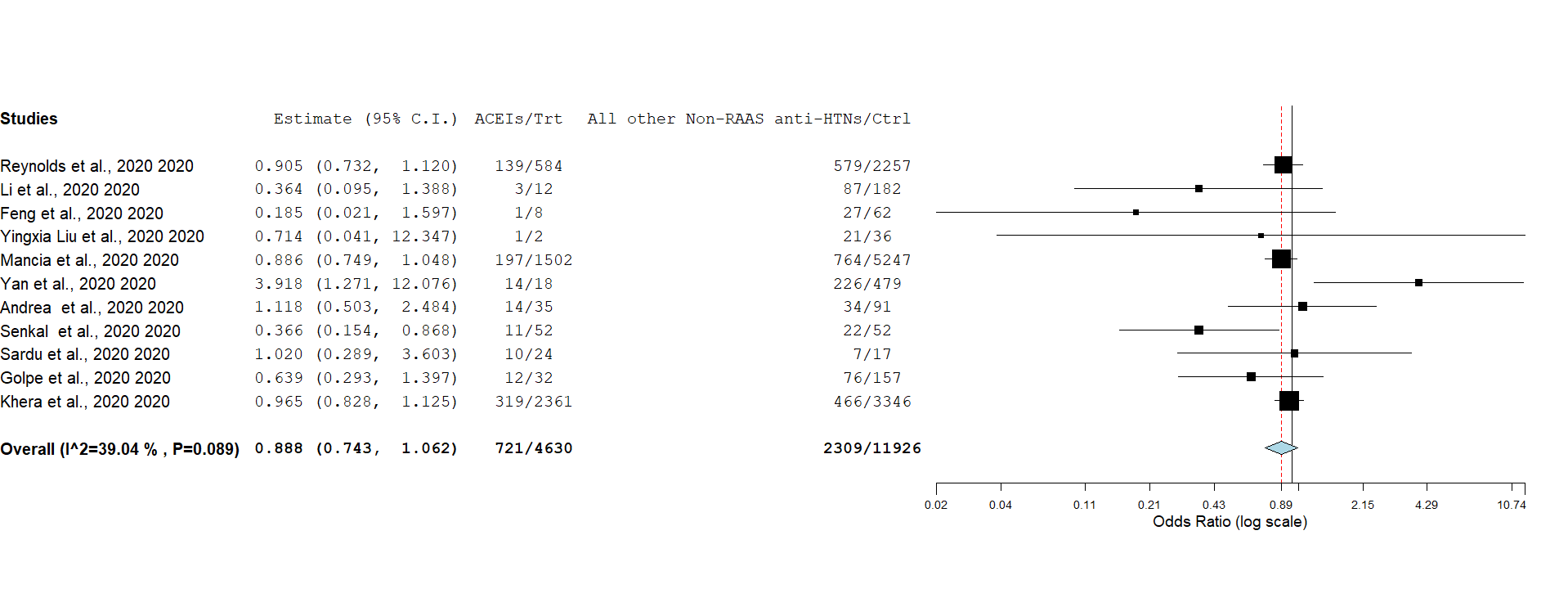


Figure S11: Risk of poor COVID-19 clinical outcome with ACEIs relative to all other non-RAAS antihypertensives.


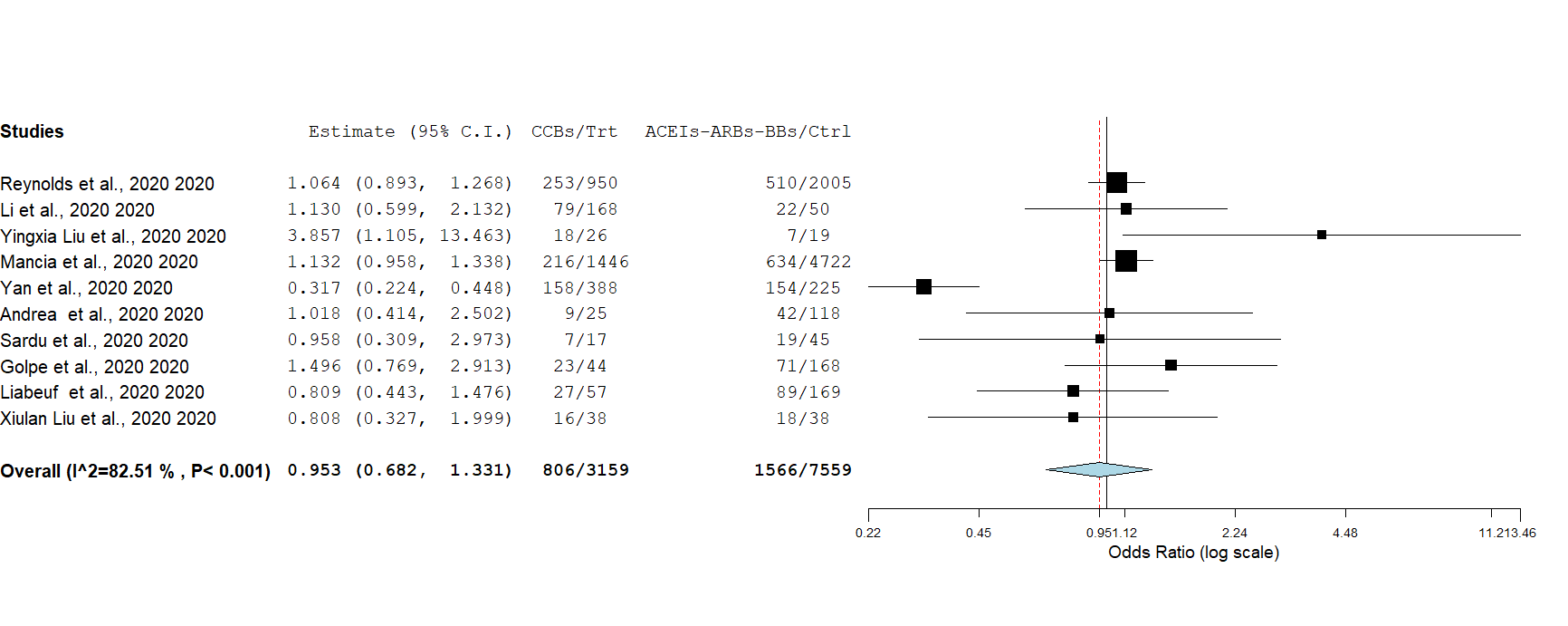


Figure S12: Risk of poor COVID-19 clinical outcome with CCBs relative to ACEI, ARBs, BBs


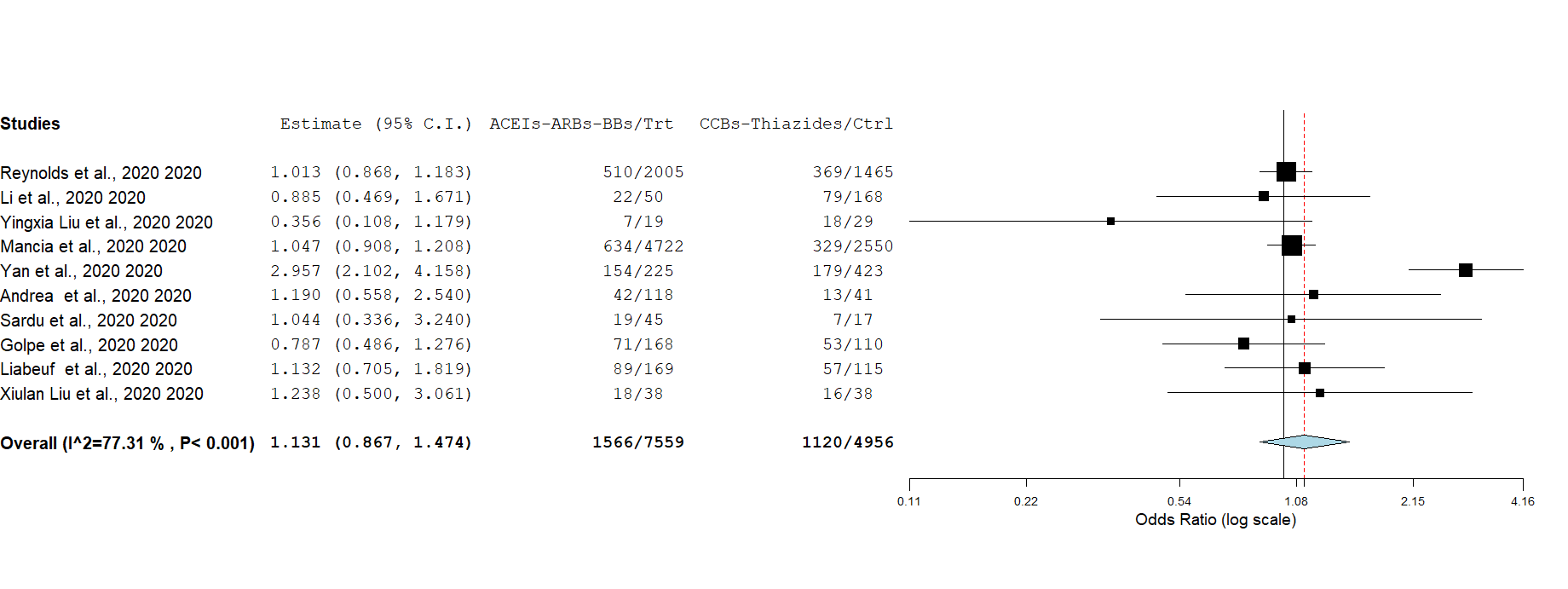


Figure S13: Risk of poor COVID-19 clinical outcome with ACEI, ARBs, BBs relative to CCBs and thiazides
